# Genes of the major histocompatibility complex highlight interactions of the innate and adaptive immune system

**DOI:** 10.7717/peerj.3679

**Published:** 2017-08-30

**Authors:** Barbara Lukasch, Helena Westerdahl, Maria Strandh, Hans Winkler, Yoshan Moodley, Felix Knauer, Herbert Hoi

**Affiliations:** 1 Department of Integrative Biology and Evolution, Konrad Lorenz Institute of Ethology, University of Veterinary Medicine, Vienna, Austria; 2 Department of Biology, Molecular Ecology & Evolution Lab, Lund University, Lund, Sweden; 3 Department of Zoology, University of Venda, Thohoyandou, Republic of South Africa; 4 Department of Integrative Biology and Evolution, Research Institute of Wildlife Ecology, University of Veterinary Medicine, Vienna, Austria

**Keywords:** Cell-mediated immunity, MHC diversity, *Passer domesticus*, Humoral immunity, Innate immunity

## Abstract

**Background:**

A well-functioning immune defence is crucial for fitness, but our knowledge about the immune system and its complex interactions is still limited. Major histocompatibility complex (MHC) molecules are involved in T-cell mediated adaptive immune responses, but MHC is also highly upregulated during the initial innate immune response. The aim of our study was therefore to determine to what extent the highly polymorphic MHC is involved in interactions of the innate and adaptive immune defence and if specific functional MHC alleles (FA) or heterozygosity at the MHC are more important.

**Methods:**

To do this we used captive house sparrows (*Passer domesticus*) to survey MHC diversity and immune function controlling for several environmental factors. MHC class I alleles were identified using parallel amplicon sequencing and to mirror immune function, several immunological tests that correspond to the innate and adaptive immunity were conducted.

**Results:**

Our results reveal that MHC was linked to all immune tests, highlighting its importance for the immune defence. While all innate responses were associated with one single FA, adaptive responses (cell-mediated and humoral) were associated with several different alleles.

**Discussion:**

We found that repeated injections of an antibody in nestlings and adults were linked to different FA and hence might affect different areas of the immune system. Also, individuals with a higher number of different FA produced a smaller secondary response, indicating a disadvantage of having numerous MHC alleles. These results demonstrate the complexity of the immune system in relation to the MHC and lay the foundation for other studies to further investigate this topic.

## Introduction

For ecologists and evolutionary biologists it is often of great importance to analyze immune responses triggered by host-parasite interactions of their study species. Host-parasite interactions have an important impact on evolution, ranging from survival and fitness within species to rate of speciation. To reveal the complex variations of immune responses among individuals, a concept called ‘immunocompetence’ was introduced as an important determinant of fitness (Altizer, Harvell & Friedle, 2003; Bernatchez & Landry, 2003). It grades the ability of a host to prevent, control and clear infections by pathogens and parasites, and its central prediction is that immune function is costly. Especially when (physiological) resources are limited, immune responses may constrain investment in other energetically costly life-history traits, such as secondary sexual traits, growth or reproduction (Lochmiller & Deerenberg, 2000; Norris & Evans, 2000; Owen, Nelson & Clayton, 2010). It has been shown that immune function varies during the annual cycle (Hegemann et al., 2013b; Pap et al., 2015) and that limited food resources can cause changes in immune function in shorebirds (Buehler et al., 2009).

It is challenging to measure and classify immune function because each immunological test can only explain a small part of the immune response. Furthermore, the knowledge about interactions within the immune system is limited, particularly so for non-model organisms. Although a multitude of assays are available, most studies have focused on only a small proportion of immunological tests. But because of its complexity, a trade-off between life-history traits and only one component of the immune system can be misleading. It is always possible that no costs are observed because compensation within the immune system has taken place or an organism can compensate (e.g., with an extra intake of food see Buehler et al., 2009; Hegemann et al., 2013a; Hegemann et al., 2013b). These examples highlight the importance of using several tests reflecting different areas of the immune system simultaneously (Buehler et al., 2011; Keil, Luebke & Pruett, 2001; Matson et al., 2006; Norris & Evans, 2000; Schmid-Hempel & Ebert, 2003).

The immune system can be divided into two main components: innate and adaptive immunity, the latter holding both cell-mediated and humoral immunity. To further understand the immune system and its complex mechanisms, immune genes might help us to reveal interactions within the immune system. In particular, genes of the major histocompatibility complex (MHC) could be suitable, because they are present in all vertebrates, they are highly polymorphic and known to play a central role in the immune system (Murphy, 2012). There are two main classes of MHC genes that are important but in this study we will only concentrate on MHC class I. These cell surface molecules typically present peptides derived from the degradation of intracellular pathogens (such as viruses and some protozoa) to cytotoxic T-lymphocytes that thereafter kill the infected host cells (Murphy, 2012). To test the impact of the MHC on the immune function, we measured immune responses (as an answer to an immune challenge) as well as baseline immune functions.

Specific MHC alleles have been shown to provide resistance/susceptibility to diseases. In captive-raised Atlantic salmon, experimental evidence for resistance of specific alleles to *Aeromonas salmonicida* bacteria (Langefors et al., 2001; Lohm et al., 2002) and to the infectious salmon anaemia virus was found (Grimholt et al., 2003). Certain MHC alleles also played a role in resistance/susceptibility to infections with gastrointestinal nematodes in captive mice (Else et al., 1990; Wassom, Krco & David, 1987) and sheep (Buitkamp et al., 1996). In wild animals, specific MHC alleles were linked to resistance/susceptibility or intensity of infection to malaria in house sparrows (Bonneaud et al., 2006; Loiseau et al., 2008), great tits (Sepil et al., 2013), blue tits (Westerdahl et al., 2013), great reed warblers (Westerdahl et al., 2012; Westerdahl et al., 2005) and to coccidiosis in the red junglefowl (Worley et al., 2010). Specific MHC alleles that were linked with survival rates have also been found in voles, house sparrows, seychelles warblers and red junglefowl (Brouwer et al., 2010; Karlsson et al., 2015; Kloch et al., 2013; Worley et al., 2010).

The aim of this study is to shed new light into the complex interactions of the innate and adaptive immune defence. Specifically we were interested to study potential relationships of functional MHC alleles (translated according to binding properties of amino acids in the peptide binding region of MHC alleles) and immune responses (as an answer to a challenge; e.g., injections of antibodies) and baseline immune functions (immunological potential of blood and plasma; measured with blood samples), which are commonly tested in wild birds and affect different areas of the immune system (innate and adaptive response). To reveal interactions within the immune system we investigated if specific alleles were linked to several immune tests and analyzed if these tests were correlated with a principal component analysis.

## Methods

### Ethics statement

All animal experiments were in accordance to the Austrian Law by the Federal Ministry of Science, Research and Economy (BMWF) (Geschäftszahl: BMWF-68.205/0081-ll/3b/2012).

We used a captive population of house sparrows (*Passer domesticus*) for several immunological tests. All of these birds were born in captivity in 2012 and housed in large flocks of the same sex in outdoor aviaries (10–15 birds/aviary; aviary size: 3.5 m × 3.5 m × 3 m) at the Konrad Lorenz Institute of Ethology in Vienna. These aviaries were equipped the same way with vegetation, perches, commercial food for granivorous passerines (ad libitum) and water. The parents of these birds were captured from the wild in Vienna, Austria in 2010 and brought into captivity. At the age of 11 days (spring 2012) and 1 year (July 2013), immune tests were conducted with these 128 one year old sparrows (52 females and 76 males) derived from 42 different families (average: 3 nestlings/family) and born in different clutches (clutch 1–4). Our aviary population of house sparrows allowed us to control for several environmental factors that might otherwise interfere with our results, like food availability (food was offered ad libitum), predation and differences in microclimate as well as socio-ecological factors including population density and age. Also, we could use a wider range of tests that require recaptures and would have been a problem in the wild.

The following immune challenge was done in 2012 when nestlings were 11 days old (*N* = 128): skin-swelling response to phytohaemagglutinin (PHA). The following immune challenges and baseline immune functions were tested in the same birds in 2013 when they were one year old (*N* = 128): hematocrit (HCT), erythrocyte sedimentation rate (ESR), bacterial killing assay (BKA) and heterophil/lymphocyte (H/L) ratio. Birds that died between the age of 11 days and one year (*N* = 60) were not used for the analysis. Due to stress and potential interdependency between tests, we had two different groups of one year old birds; we tested first and second skin-swelling response to PHA in group 1 and haemagglutination titers after sheep red blood cell (SRBC) injections in group 2 (see Table 1 for experimental design). We chose these tests and surveys because they correspond to the innate and the adaptive (cell-mediated and humoral) immune responses (Table 2, see supplement for further details of immune tests). Since longer handling stress could affect different immunological measurements, blood was taken within 20 min after capture (Buehler et al., 2008). For each bird, we collected blood samples from the brachial vein (100 µl per bird) into sterile, heparinized capillary tubes. The immunological tests were performed prior to MHC screening and therefore blindly with respect to individuals’ genotypes. We additionally measured tarsus length and body mass to calculate a condition factor (residuals of weight/tarsus). All animal experiments were in accordance to the Austrian Law (Geschäftszahl: BMWF-68.205/0081-ll/3b/2012).

**Table 1:** Schematic presentation of the experimental design.

Schedule	Group 1 + 2 (*N* = 128)	Group 1 (*N* = 64)	Group 2 (*N* = 64)
	(*M* = 76, *F* = 52)	(*M* = 31, *F* = 33)	(*M* = 45, *F* = 19)
2012 (Spring)			
- Day 0	PHA injection (nestlings) + Body measurements		
- Day 1	PHA response		
2013 (July)			
- Day 0	Blood (100 µl) + Body measurements + HCT + ESR + H/L + BKA	1st PHA injection	SRBC injection
- Day 1		1st PHA response	
- Day 7		2nd PHA injection + Body mass	
- Day 8		2nd PHA response	Blood (100 µl) + Body mass + SRBC (Agglutination)

**Table 2:** Immune tests used correspond to the innate and adaptive (cell-mediated and humoral) immune response.

Immune tests	Age class (year)	Innate immunity	Adaptive immunity	
			Cell-mediated	Humoral
HCT	1	X		
ESR	1	X		
H/L	1	X	X	
BKA	1	X		
PHA (nestling)	0	X	X	
PHA (1st response)	1	X	X	
PHA (2nd response)	1	X	X	
SRBC	1			X

### Molecular methods

Avian blood was stored in 95% ethanol and DNA was extracted using the DNeasy Blood & Tissue Kit (Qiagen) according to the manufacturer’s instructions.

#### MHC genotyping

Characterization of the MHC class 1 exon 3, encoding parts of the peptide binding region, was carried out using 454 amplicon sequencing, according to Galan et al. (2010), refined for house sparrows by Karlsson & Westerdahl (2013). Sequence coverage was aimed at 300 reads per individual (according to Borg et al., 2011). Four hundred ninety-one individuals were run in two different sequencing reactions (run 1 (R1) and run 2 (R2)) and these runs were filtered separately (see below). R1 contained 272 samples (243 individuals + 29 technical replicates) and R2 contained 286 samples (248 individuals + 38 technical replicates). 454 sequencing was done at Lund University Sequencing Facility (Faculty of Science), Sweden.

#### MHC - bioinformatics and data processing

After 454 sequencing, the data was extracted and assigned to samples using the program jMHC (Stuglik, Radwan & Babik, 2011). We had a total of 75,894 sequence reads of 19,200 unique sequences in 454 R1 that had complete tags and primers. In R2 we had a total of 125,752 sequence reads of 34,670 unique sequences. To discard potential artifacts generated during the initial PCR, the emulsion PCR and the 454 sequencing reaction the raw data from the 454 run were filtered for low abundance (alleles with <3 reads in the total dataset were removed), sequence coverage (minimum 104 reads/individual), low frequency per individual (alleles with lower prevalence than 2% were removed), independence (alleles must occur in at least two independent PCRs), chimeras and singleton substitutions according to Zagalska-Neubauer et al. (2010) and Sepil et al. (2012). See supplement for further details. The filtered MHC DNA sequences (*N* = 85) were translated into amino acid sequences (AA, *N* = 78). From these sequences “functional MHC alleles” (FA,  *N* = 59) were identified based on the chemical binding properties of the amino acids in the peptide binding region. In detail, the 16 amino acids from each sequence corresponding to the peptide binding region in *Gallus gallus* (Wallny et al., 2006) were extracted and converted into five physicochemical descriptor variables: *z*1 (hydrophobicity), *z*2 (steric bulk), *z*3 (polarity), *z*4 and *z*5 (electronic effects) (Sandberg et al., 1998). MHC diversity was evaluated as: (1) the number of different FA per individual and (2) presence/absence of the 10 most common FA (all of which occurred in more than 10% of the individuals).

#### Neutral genetic variation

For the assignment of neutral individual heterozygosity, all individuals were typed at 13 highly polymorphic neutral microsatellite loci. The quality of loci (Hardy-Weinberg Equilibrium and Linkage Disequilibrium) was tested using the programs Cervus (v3.0.3) and Fstat (v2.9.3.2). Individuals were genotyped in two multiplex reactions according to the protocol of Dawson et al. (2012) (Table S1). The loci used in this protocol are known to be distributed across at least seven different chromosomes and thus likely reflect the genome-wide level of neutral genetic variation (Dawson et al., 2012). Allele sizes were determined using GeneMapper (v3.0) with reference to an in-house DNA size standard (HMROX). We successfully amplified 12 loci (Table S1). However, we excluded locus Pdoµ6 due to amplification problems.

### Statistical analysis

#### Treatment effect on weight

Since we had two different treatment groups (PHA or SRBC injections), we tested if there was a treatment effect on body mass. Weight was measured on day 0, and at day 7 and 8 in group 1 and 2, respectively, to see if the different treatments influenced weight after one week. We conducted a linear mixed model within the R package v.3.0.3 x64 (R Core Team, 2013) with sex and treatment group as fixed factors, family origin as random effect and difference in weight as dependent variable. Non significant results were removed sequentially.

#### Relationship between MHC and the immune system

We used linear mixed models within the R package v.3.0.3 x64 (R Core Team, 2013) to analyze the relationship between MHC and several immunological parameters. ESR, HCT, BKA, H/L ratio, PHA (nestlings and first and second response after one year) and SRBC were used as dependent variables in different models. The number of different functional MHC alleles, presence/absence data of the ten most common functional alleles, microsatellite heterozygosity, sex, clutch number and body condition were included as fixed factors and family origin was used as a random effect. In order to evaluate and compare different models, we used the Akaike Information Criterion corrected for small sample sizes (AIC_c_, Burnham & Anderson (2002)). In all cases there was not one clearly best model, so we used methods of model averaging and multimodel inference (Burnham & Anderson, 2002). These methods allow inference over all models considered, but this was weighted according to model support by the data. Additionally, these methods do not only estimate standard errors unconditionally for a single model, they also provide the probability for single variables being in the unknown “true” model (the so-called relative variable importance - RVI). Unlike variable selection based on *p*-values these techniques have a sound mathematical basis and are increasingly recommended (Symonds & Moussalli, 2011). We conducted these calculations in R using the package MuMIn (Barton, 2013). Additionally, we inspected the residuals for normality visually using histograms and QQ-plots. H/L ratio and ESR were log-transformed to achieve normality. Since we included the 10 most common functional MHC alleles in our analysis (frequency on the population level >10%), we had to do a pre-model selection of variables. In the first model we evaluated the relevance of the 10 alleles, while all other factors (number of functional MHC alleles, microsatellite heterozygosity, clutch number and condition) were fixed.

We then included only functional MHC alleles in the final model that had RVI values larger than 0.5. In the results section, only variables that have RVI values >0.7 are discussed.

#### Covariance of different immune measures

We conducted a principle component analysis (PCA) with individuals of group 1 (*N* = 64) to gain insight into the complex relationships within different immune measures. It is a method to derive linear combinations of original variables in a dataset to summarize variation. Covariance can be identified among more than two variables and this method is increasingly used to summarize data taken from multiple measures of immune function (Buehler et al., 2011; Matson et al., 2006). We used varimax rotation to maximize contrasts of variable loadings between factors. We were especially interested in relationships of the immune system for individuals of group 1, since we wanted to see the relationship of the repeated PHA measurements.

## Results

### MHC characterization

**Figure 1:**
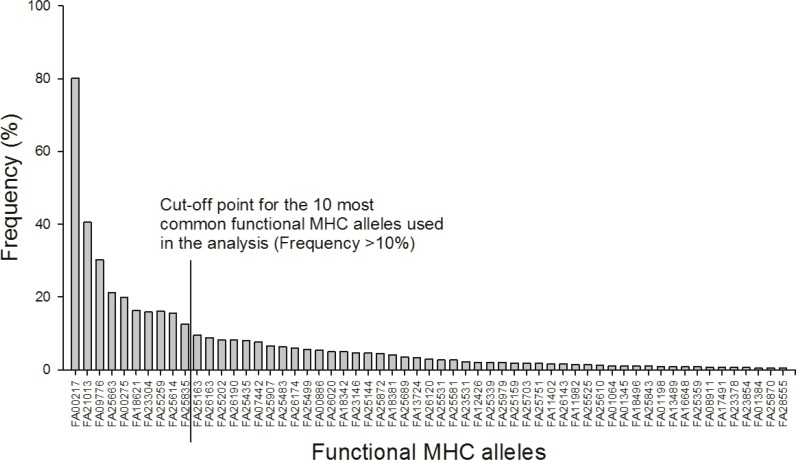
Frequency distribution of the 59 functional MHC class I alleles and cut-off point for the ten most common alleles used in the analysis (Frequency >10%).

A total of 85 different MHC alleles were found in 491 individuals and these could be translated into 59 unique functional MHC alleles (FA; Tables S2 and S3). There was on average 4.2 FA per individual (ranging from 1–8; 4.2 alleles (sd: 1.4) in males and 4.3 (sd: 1.4) in females). Ten FA occurred in more than 10% of the individuals and these were included in the downstream analyses (Fig. 1).

### Treatment effects on weight

There was a significant difference in weight change following treatment after one week between the two groups (RV I = 0.77, *p* = 0.034). The weight of the individuals in group 2 (SRBC injections) was stable (*N* = 64, *MW* + 0.06 g, sd: 0.90), whereas the individuals in group 1 (PHA injections) gained weight after the first PHA injection (*N* = 64, *MW* + 0.45 g, sd: 0.99). Neither sex nor the interaction of sex and treatment group were significant.

#### Relationship between MHC and innate immunity

**Figure 2:**
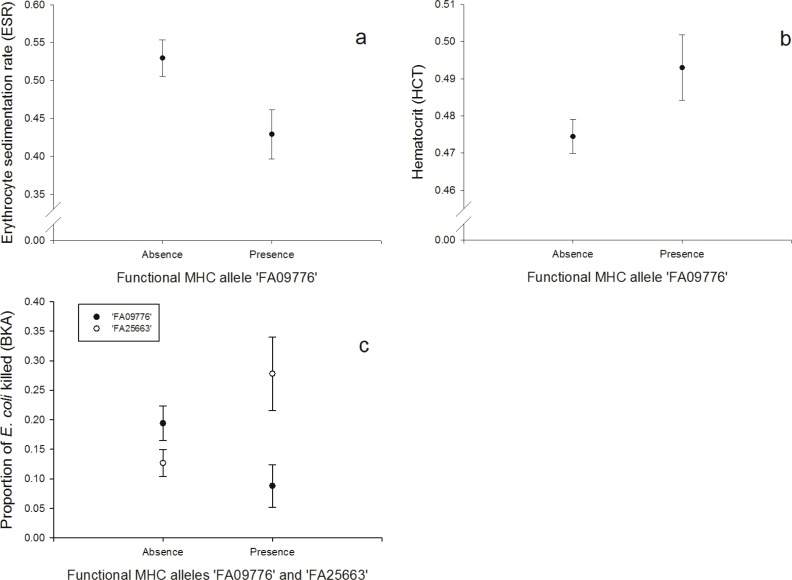
Innate immunity: (A) Relationship between the FA ‘FA09776’ and ESR (log-transformed), (B) Relationship between the FA ‘FA09776’ and HCT, (C) Relationship between the FAs ‘FA09776’ and ‘FA25663’ and BKA.

One FA, ‘FA09776’, was negatively correlated with the response to the erythrocyte sedimentation rate (ESR, *N* = 128; Fig. 2A, Table S4) and the bacterial killing assay (BKA, *N* = 128; Fig. 2C, Table S5), whereas it was positively correlated with hematocrit (HCT, *N* = 128; Fig. 2B, Table S6). FA ‘FA25663’ had a reversed association with the BKA response, hence it was positively correlated (*N* = 128; Fig. 2C, Table S5).

**Figure 3:**
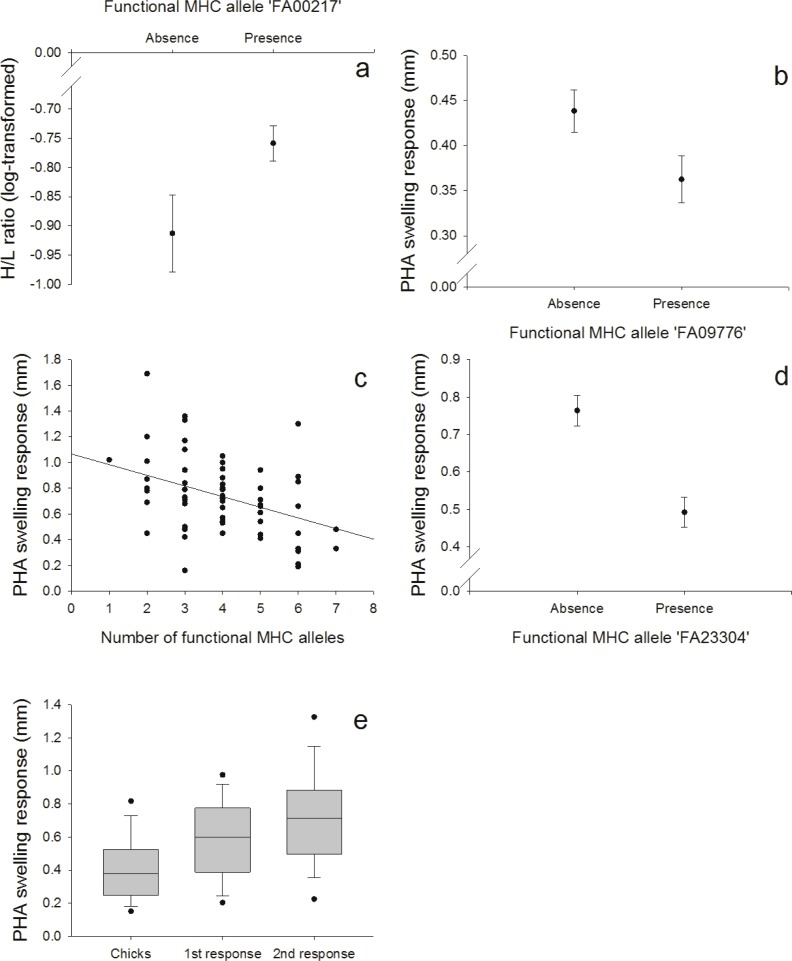
Innate and adaptive (cell-mediated) immunity: (A) Relationship between the FA ‘FA00217’ and H/L ratio (log-transformed), (B) Relationship between the FA ‘FA09776’ and PHA response in nestlings, (C) Relationship between the number of different FA per individual and the second PHA response in adults, (D) Relationship between the FA ‘FA23304’ and the second PHA response in adults, (E) PHA response in nestlings and adults (first and second response).

#### Relationship between MHC and innate and adaptive (cell-mediated) immunity

We found a positive correlation between the FA ‘FA00217’ and the heterophil/lymphocyte (H/L) ratio (*N* = 128; Fig. 3A, Table S7). Skin-swelling response to phytohaemagglutinin (PHA) was tested in both nestlings and adults. In nestlings (*N* = 128), PHA was negatively correlated with the FA ‘FA09776’ (Fig. 3B, Table S8). In adults (*N* = 64), the first PHA response was not correlated with MHC (Table S9), but the second response (measured one week later) was negatively correlated with both the number of different FA per individual (Fig. 3C, Table S10) and the FA ‘FA23304’ (Fig. 3D, Table S10). In adults, second swelling response to PHA was higher than first response (paired *t*-test: *p* < 0.001); first response in adults was higher than response in nestlings (paired *t*-test: *p* < 0.001) (Fig. 3E). This indicates an adaptive response to repeated PHA injections leading to a larger swelling reaction.

#### Relationship between MHC and adaptive (humoral) immunity

**Figure 4:**
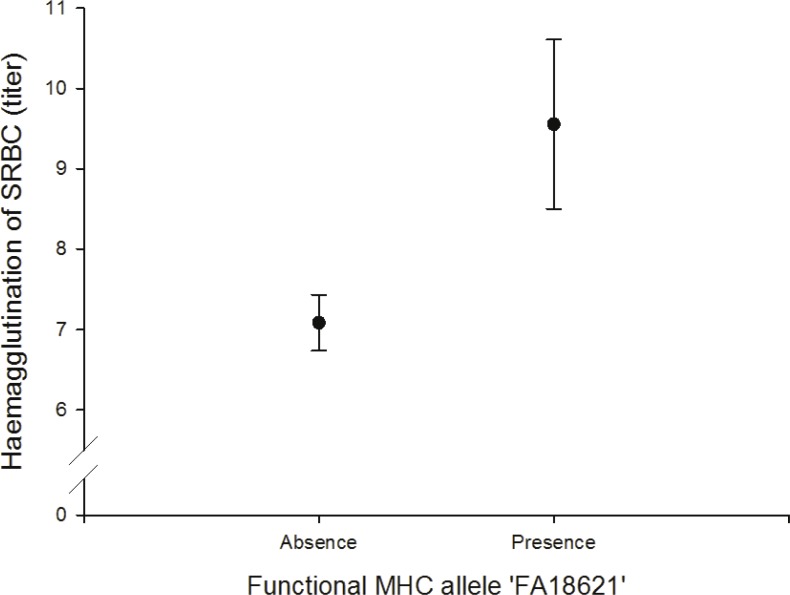
Adaptive (humoral) immunity: relationship between the FA ‘FA18621’ and SRBC.

We found a positive correlation between the FA ‘FA18621’ on agglutination of sheep red blood cells (SRBC) (*N* = 64; Fig. 4, Table S11).

#### Covariance of different immune measures

Our principal component analysis identified three PCs with eigenvalues > 1 that cumulatively accounted for 63% of the total variation (Table 3). The patterns of loadings on these three PCs revealed that ESR, HCT and H/L correlated with PC1 (26.2% of total variation), PHA in nestlings and BKA correlated with PC2 (18% of total variation) and PHA first and second response in adults correlated with PC3 (18% of total variation).

## Discussion

In this study we show that several of the functional MHC alleles were linked to responses of the innate and adaptive immunity.

### Innate immunity

Especially one FA was linked to all of the immune tests that measured responses of the innate immunity (HCT, ESR, BKA, PHA in nestlings, *N* = 128). Individuals possessing the allele ‘FA09776’ had a lower ability to kill *E. coli* bacteria *in vitro* (BKA), a lower ESR, a lower swelling response to PHA in nestlings, but a higher HCT. The significant ‘FA09776’ occurred in a high frequency (32.81%) and was the third most common FA in our population. The ESR is increased during any cause of inflammation and the HCT reflects blood oxygen-carrying capacity; it can be low in acute or chronic diseases, nutritional deficiencies, parasite infested individuals and bacterial infections (review: Fair, Whitaker & Pearson, 2007). According to that, ‘FA09776’ would be beneficial for individuals in terms of a lower ESR and a higher HCT. But on the other hand, it was disadvantageous for the BKA (less *E. coli* killed) and PHA in nestlings (lower swelling response). These results highlight that general conclusions about advantages or disadvantages of specific MHC alleles based on immune responses of single tests are hard to predict, since we only have limited insights in the complex interactions within the immune system and our knowledge about the underlying physiological mechanisms and relationships are limited.

**Table 3:** Principal-component loadings after varimax rotation for seven different measures of innate and adaptive immunity (group 1, *N* = 64). ESR, HCT and H/L correlate with PC1 (26.2% of total variation), PHA in nestlings and BKA correlate with PC2 (18% of total variation) and PHA first and second response in adults correlate with PC3 (18% of total variation).

	PC1	PC2	PC3
PHA (nestlings)	−0.022	**0.807**	0.006
HCT	**−0.777**	0.104	−0.207
H/L	**0.674**	−0.216	−0.269
BKA	0.038	**−0.756**	0.051
PHA (adults) 1st response	0.136	−0.164	**0.726**
PHA (adults) 2nd response	−0.074	0.103	**0.791**
ESR	**0.835**	0.193	0.071
Variance per component (%)	26.238	18.741	18.055
Cummulative variance (%)	26.238	44.978	63.034

A PCA revealed that ESR and HCT (both associated with the same FA ‘FA09776’) were also correlated together and with the H/L ratio (PC1), hence PC1 might reflect measurements of blood parameters. Similarly, BKA and PHA in nestlings were also correlated (PC2). Thus PC2 may be interpreted as an innate response factor, which is supported by the fact that *E. coli* is primarily attacked by soluble blood components rather than phagocytosis. The BKA assay is a useful tool to broadly characterize an individual’s capacity to prevent infections once they have reached the bloodstream. *E.coli* bacteria commonly infect house sparrows and their plasma contains proteins (globulins) and lysozyme (enzyme) that can fight these infections (Millet et al., 2007). While individuals with the FA ‘FA09776’ (that also linked with ESR, HCT and PHA in nestlings), had lower rates of *E. coli* bacteria killed, the FA ‘FA25663’ had a contrary effect and individuals with this allele had higher rates of *E. coli* bacteria killed.

### Innate and adaptive (cell mediated immunity)

Especially in our 11 days old nestlings we expected a more innate response to the PHA injections. This prediction was supported by the fact that the PHA swelling response in nestlings was correlated with the BKA, a test of the innate immunity (PC2), but not with the two PHA injections in adults that were correlated and explained another component of the PCA (PC3). Also, we found that the FA ‘FA09776’ that was linked with three measures of the innate immunity (ESR, HCT and BKA), was also related with the PHA swelling response in nestlings. On the contrary, we found that the second PHA response in adults was linked to another FA (‘FA23304’). This indicates that first and second response to PHA in adults and PHA response in nestlings might not measure the same area of the immune system. In nestlings, swelling response might depend more on the innate response, in adults, adaptive immunity could be more important. The PHA skin-swelling test is a robust tool for a quick screening of an individuals’ proinflammatory potential (i.e., the ability to mount an inflammatory response; Tella et al., 2008; Vinkler, Bainova & Albrecht, 2010). In a meta-analysis, Møller & Saino (2004) showed that survival of different bird species was positively correlated with PHA responses. The swelling response is highly complex, involving both cells of the innate and adaptive (cell-mediated) immunity (Martin II et al., 2006).

Although the number of different FA per individuals had no effect on the primary response to PHA in adults, individuals possessing a higher number of different FA produced a smaller secondary response to the mitogen, indicating a disadvantage of having numerous MHC alleles. Individuals heterozygous at a MHC locus should theoretically recognize twice as many foreign peptides as homozygotes (Hughes & Nei, 1992; Lenz et al., 2013; Milinski, 2006), and it is therefore surprising to find that individuals with higher MHC diversity (more different alleles/individual) had a weaker response against the second PHA injection. These results could indicate that the expression of numerous alleles might be costly, but again, a general conclusion based on this immune response is hard to predict. In our population, one year old birds in group 1 (PHA injections) gained weight after the first PHA injection. Immunological challenges or disease (*e.g.,* anemia or parasites) could lead to a higher food intake and might mask potential negative fitness effects because individuals might try to compensate for the higher physiological needs with higher food intake possibly resulting in weight gain (Norris & Evans, 2000). PHA treatment has already been shown to increase food intake in birds (Barbosa & Moreno, 2004; Martin II, Scheuerlein & Wikelski, 2003).

The H/L ratio has widely been used as an indicator of stress in birds. In response to stressors, the relative number of heterophils in peripheral blood increases while the relative number of lymphocytes decreases (Gross & Siegel, 1983). It can be increased during times of low food availability, in parasite-infested, migrating or breeding birds (review by Davis, Maney & Maerz, 2008) and has been used as an indicator of the immune function (Hegemann et al., 2012; Krams et al., 2012; Matson et al., 2012). High H/L ratios might also lead to a depression of some immune indices (Krams et al., 2012; Müller, Jenni-Eiermann & Jenni, 2011) and can cause higher mortality rates (Kilgas et al., 2006; Ochs & Dawson, 2008). In our population, individuals with the FA ‘FA00217’, the most common allele (80.04%), had higher H/L ratios, indicating a disadvantage of this allele or something linked to it in terms of higher stress levels.

### Adaptive (humoral) immunity

*In vivo* humoral immune response was assessed by measuring primary antibody response to intraperitoneal injections of SRBC. It gives information about the antibody-mediated immunity (IgM and IgG). Like skin tests, measurement of antibody titers following immunization with an antigen integrates a large number of immunological functions and events. If sufficient antibody activity is present, the antibodies will agglutinate the SRBC, which causes a visible spreading of the SRBC on the bottom of the wells. We found that the FA ‘FA18621’ was linked to the SRBC agglutination test and individuals with this allele had higher antibody activity to agglutinate the SRBC. Other studies in house sparrows and chicken also found a relationships between specific MHC alleles and SRBC (Bonneaud et al., 2005; Dunnington et al., 1984).

## Conclusions

With our aviary population, we were able to show different interactions within the immune system in relation to MHC diversity, while other factors could be controlled for, highlighting the genetic influence. We especially focused on functional MHC diversity which is based on properties of amino acids in the peptide binding region. We found that different functional MHC alleles and the number of different functional MHC alleles were linked to different areas of the innate and adaptive immunity, reflected by: HCT/ESR, BKA, H/L ratio, PHA and SRBC. These results help to shed new light into the complex interactions of the innate and adaptive immune defence and lay a foundation for further studies to investigate this topic.

##  Supplemental Information

10.7717/peerj.3679/supp-1 Supplemental Information 1 Supplementary Methods: Immune tests; MHC - bioinformatics and data processing; Suppl. Tables 1-11; Supplementary literature

## Additional Information and Declarations

### DNA Deposition

The following information was supplied regarding the deposition of DNA sequences: Genbank accession numbers of the 65 novel MHC class I exon 3 alleles found in this study: Pado-UA_253, 261-299 and 328-352; provided in Table S2.

### Data Availability

The following information was supplied regarding data availability: Raw data deposited with Dryad: 454 sequence data and all information needed to demultiplex the raw data to assign each read to a sample, the microsatellite genotype data, the aligned MHC sequences for all loci and samples and the aligned functional MHC alleles for all loci and samples. Dryad DOI 10.5061/dryad.5m6t4.
