# Supplementary material for: Genes of the major histocompatibility complex highlight interactions of the innate and adaptive immune system
Source: PeerJ. 2017 Aug 30;5:e3679. doi: 10.7717/peerj.3679 (PMC5581531; doi:10.7717/peerj.3679)
Supplement: Supplemental Information 1 [file peerj-05-3679-s001.docx]

**Supplementary Material**

**Supplementary Methods**

**Immune tests**

***Erythrocyte sedimentation rate (ESR) / Hematocrit (HCT)*:** To measure the immunological structure we analyzed ESR and HCT (in duplicate), these are standard methods used for hematological parameters ([Schalm, Jain & Carroll 1975](#_ENREF_67)). We took fresh blood samples (about 20 µl) from the brachial vein and collected the blood into heparinized capillary tubes and immediately sealed them with wax at the bottom. The capillary tubes were positioned vertically for 4 h at 4°C. ESR was measured with a digital caliper to the nearest 0.01 mm and calculated as the distance occupied by plasma divided by the total length of the blood column (red blood cells + plasma). Capillary tubes were centrifuged for 10 min at 12,000 g and HCT was measured as the relative amount of red blood cells divided by total blood volume (red blood cells + plasma) with a digital caliper the nearest 0.01 mm.

***Bacterial killing assay (BKA)*:** We measured constitutive innate immunity by assessing the capacity of plasma to kill microorganisms in vitro for gram-negative bacteria (*Escherichia coli*), using spectrophotometry ([according to Liebl & Martin II 2009](#_ENREF_44)). Plasma was extracted by centrifuging fresh blood at 12,000 g for 10 min at 4°C and collected into new sterile 1.5 µl microcentrifuge tubes. Plasma samples were frozen at -80°C within 2 h and analyzed in triplicate within 5 days after collection. *E.coli* bacteria (American Type Culture Collection ATCC8739, Microbiologics Inc.), supplied as lyophilized pellets (6.6 x 10⁷ colony forming units (CFU) per pellet) were reconstituted per manufacturer instructions in 10 ml sterile PBS (phosphate buffered saline, P4417 Sigma-Aldrich). Before each assay, microbial stocks were diluted to a working concentration of 1x10⁵ microbes/ml). Plasma was diluted 1:23 with sterile PBS (1.5 µl plasma with 34.5 µl PBS) and microbial working solution (12.5 µl) was added, vortexed and incubated (37°C) for 30 min. All procedures were performed in a laminar flow hood, providing a sterile working environment in the laboratory. After incubation, all samples were removed, vortexed and 250 µl of sterile TSB (tryptic soy broth, 22092 Sigma-Aldrich) was added to each sample. Broth was vortexed again and incubated (12 h at 37°C). The number of microbes in the initial inoculum served as a positive control (12.5 µl working culture + 36 µl PBS + 250 µl of TSB). Solutions of 250 µl sterile TSB and 48.5 µl sterile PBS were used as blanks. Microbial concentrations were determined by measuring the absorbance of each sample at 300 nm using a Nanodrop. The anti-microbial activity of plasma and blood was calculated as 1-(absorbance of sample⁄absorbance of control), or the proportion of microbes killed in samples relative to positive controls.

***Heterophil/Lymphocyte (H/L) ratio*:** Circulating cellular immunity was assessed using differential leucocyte counts of heterophils and lymphocytes by examining blood smears. Blood slides were prepared within 2 min after the blood was taken using the standard two slide wedge procedure ([Campbell 1995](#_ENREF_14)). All samples were air-dried and fixed with absolute methanol and stained with Giemsa (Roth, T862.1). Differential leucocyte counts of 100 heterophils or lymphocytes were carried out using a light microscope (1000× magnification with oil immersion).

***Skin-swelling response to phytohaemagglutinin (PHA)*:** The PHA skin-swelling test was used to estimate cell-mediated immunity and to screen proinflammatory potential of individuals ([Tella *et al.* 2008](#_ENREF_75); [Vinkler, Bainova & Albrecht 2010](#_ENREF_77)). Following assay was used ([according to Bonneaud *et al.* 2005](#_ENREF_6)): The left wing web was injected with 0.02 mg of PHA-P (Sigma-Aldrich: L8754) in 0.04 ml of PBS, while the right wing web was injected with 0.04 ml of PBS as a control. Thickness of swelling was measured 24 h after immunization with a pressure-sensitive spessimeter to the nearest of 0.01 mm. Intensity of the response was assessed as the difference in swelling between the PHA injected and the control wing. This was done for 11 days old nestlings and adults. Adults received two injections, the second PHA injection seven days after the first.

***Haemagglutination of sheep red blood cells (SRBC)*:** In vivo humoral immune response was assessed by measuring primary antibody response to intraperitoneal injections of a suspension of sheep red blood cells (Sigma-Aldrich: R3378), with a standard haemagglutination method ([Pap *et al.* 2008](#_ENREF_64)). We took a blood sample and injected birds with 100 μl of a 10% SRBC suspension. After 8 days we took another blood sample to assess the primary response to SRBC. After centrifugation, plasma was stored at -80°C for less than one month. We estimated the production of anti-SRBC antibodies using the haemagglutination method using duplicates. The plasma was heated to 56°C for 30 min to prevent lysis of red blood cells by the complement. After pipetting 15 μl sterile PBS in each well of a U-shaped 96-well microtitre plate, 15 μl plasma was pipetted into the first column. Plasma was then serially diluted in PBS (dilution: 1/2 to 1/2048 with one negative control (PBS) per row). We then added 15 μl of a 1% SRBC suspension to these dilutions. After agitation, the plates were covered by parafilm and incubated at 37°C for 60 min. Agglutination was visually determined and when sufficient antibody activity was present, antibodies agglutinated the SRBC. Titers were expressed as the log2-transformed reciprocal of the highest dilution of plasma showing positive haemagglutination.

**MHC - bioinformatics and data processing**

First, sequences in low abundances (<3 reads) were deleted. Identical sequences within individuals were detected and merged using the web-applications ‘seqeqseq’ (http://mbio-serv2.mbioekol.lu.se/apps/seqeqseq.html) and ‘mergeMatrix’ (http://mbio-serv2.mbioekol.lu.se/apps/mergeMatrix.html).

Second, we only retained sequences with a suitable sequence depth of coverage. Since the maximum number of classical MHC alleles in house sparrows is eight, we chose a threshold of 104 reads per sample, thus giving a genotype score of 99.9% (m=8, Galan *et al.* 2010). Here we used the web-application ‘popMatrix’ (http://mbio-serv2.mbioekol.lu.se/apps/popMatrix.html) and filter 1 (from a selection of four filters).

Third, true alleles are likely to occur more frequently than artifact alleles. Therefore we used our replicates to set these thresholds in R1 and R2, deleting all alleles that occurred at lower than 2% per sample using filter 3.

Fourth, to verify true MHC alleles we determined that they should occur in at least two independent PCRs. However, since we worked with pedigree data (189 adults produced about 300 offspring, individuals were distributed across R1 and R2), every allele is therefore likely to occur several times. Therefore we used the criteria that every allele should be observed at least twice, here we used filter 4.

Fifth, sequences thus obtained were examined in BioEdit (v7.2.0) and those not matching the expected length of 222-225 bp or displaying indels that were not multiples of three base pairs were deleted. Next, PCR recombinants (chimeras) and 1-bp substitutions were removed by examining all sequences starting with those with the lowest population level frequencies (Sepil, Lachish & Sheldon 2013). All sequences occurring at a frequency below 2% were checked by eye, as were 50% of the sequences between a frequency of ≥ 2% to 25%, but no chimeras or 1-bp substitutions were observed. Therefore, we assume that sequences with a frequency of ≥ 2% were true alleles.

Supplementary Table 1: Neutral genetic variation

| Primer set | PCR mix | Size range | Number of alleles/locus | Primer set reference |
| --- | --- | --- | --- | --- |
| Ase 18 | 1 | 185-249 | 17 | Richardson et al. (2000), see Griffith et al. (2007) |
| Pdoµ1 | 1 | 156-200 | 18 | Neumann & Wetton (1996) |
| Pdoµ3 | 1 | 113-181 | 18 | Neumann & Wetton (1996) |
| Pdoµ5 | 1 | 202-264 | 21 | Griffith et al. (1999) |
| Pdoµ6* | 1 |  |  | Griffith et al. (1999) |
| Pdo9 | 1 | 362-424 | 18 | Griffith et al. (2007) |
| Pdo10 | 1 | 102-152 | 14 | Griffith et al. (2007) |
| Pdo16A | 2 | 270-302 | 15 | Dawson et al. (2012) |
| Pdo17 | 2 | 192-244 | 22 | Dawson et al. (2012) |
| Pdo19 | 2 | 173-183 | 4 | Dawson et al. (2012) |
| Pdo22 | 2 | 92-130 | 17 | Dawson et al. (2012) |
| Pdo27 | 2 | 224-248 | 12 | Dawson et al. (2012) |
| Pdo40A | 2 | 298-328 | 15 | Dawson et al. (2012) |

* had to be excluded due to amplification problems

Supplementary Table 2: Allele frequencies and GenBank Acc nr of the MHC class I exon 3 alleles found (range of the 65 novel alleles from this study: Pado-UA_253-352)

| Sequence identity from the program ‘seqeqseq’ | MHC allele names | Genbank Acc nr (NCBI BLAST) | Frequencies (%) |
| --- | --- | --- | --- |
| seq00217 | Pado-UA_238 | KJ825933* | 58.90 |
| seq21013 | Pado-UA_239 | KJ825934* | 35.79 |
| seq25566 | Pado-UA_251 | KJ825942* | 29.45 |
| seq25663 | Pado-UA_245 | KJ825936* | 21.27 |
| seq25614 | Pado-UA_338 | KP940330 | 15.75 |
| seq25401 | Pado-UA_299 | KP940319 | 14.52 |
| seq25986 | Pado-UA_244 | KJ825935* | 14.31 |
| seq00275 | Pado-UA_261 | KP940280 | 12.88 |
| seq25835 | Pado-UA_343 | KP940335 | 12.47 |
| seq25361 | Pado-UA_246 | KJ825937* | 11.45 |
| seq26078 | Pado-UA_248 | KJ825939* | 10.43 |
| seq25163 | Pado-UA_322 | JN609643.1* | 9.61 |
| seq26163 | Pado-UA_257 | KJ825947* | 8.79 |
| seq25202 | Pado-UA_297 | KP940317 | 8.38 |
| seq26190 | Pado-UA_267 | KP940286 | 8.18 |
| seq25435 | Pado-UA_329 | KP940321 | 8.18 |
| seq07442 | Pado-UA_256 | KJ825946* | 7.57 |
| seq25764 | Pado-UA_342 | KP940334 | 7.36 |
| seq08183 | gb\|KC585634.1\| | KC585634.1* | 7.16 |
| seq25459 | Pado-UA_330 | KP940322 | 6.54 |
| seq25907 | Pado-UA_258 | KJ825948* | 6.54 |
| seq25422 | Pado-UA_328 | KP940320 | 6.34 |
| seq25483 | Pado-UA_331 | KP940323 | 6.34 |
| seq24170 | Pado-UA_294 | KP940314 | 5.93 |
| seq04612 | Pado-UA_269 | KP940288 | 5.93 |
| seq25499 | Pado-UA_332 | KP940324 | 5.52 |
| seq26010 | Pado-UA_346 | KP940338 | 5.52 |
| seq00886 | Pado-UA_262 | KP940281 | 5.32 |
| seq25331 | Pado-UA_298 | KP940318 | 5.32 |
| seq26020 | Pado-UA_347 | KP940339 | 5.11 |
| seq26092 | Pado-UA_349 | KP940341 | 5.11 |
| seq25144 | Pado-UA_295 | KP940315 | 4.70 |
| seq25872 | Pado-UA_319 | JN609642.1* | 4.50 |
| seq18381 | Pado-UA_284 | KP940304 | 4.09 |
| seq25416 | Pado-UA_254 | KJ825944* | 3.89 |
| seq07562 | Pado-UA_253 | KP940294 | 3.68 |
| seq25677 | Pado-UA_339 | KP940331 | 3.48 |
| seq25689 | Pado-UA_340 | KP940332 | 3.48 |
| seq13724 | Pado-UA_281 | KP940301 | 3.27 |
| seq26130 | Pado-UA_350 | KP940342 | 3.27 |
| seq04517 | Pado-UA_268 | KP940287 | 3.07 |
| seq22920 | Pado-UA_289 | KP940309 | 2.86 |
| seq06551 | Pado-UA_272 | KP940291 | 2.66 |
| seq25531 | Pado-UA_334 | KP940326 | 2.66 |
| seq25581 | Pado-UA_250 | KJ825941* | 2.66 |
| seq18342 | Pado-UA_283 | KP940303 | 2.45 |
| seq26154 | Pado-UA_352 | KP940344 | 2.45 |
| seq23531 | Pado-UA_292 | KP940312 | 2.25 |
| seq12426 | Pado-UA_279 | KP940299 | 2.04 |
| seq18845 | Pado-UA_287 | KP940307 | 2.04 |
| seq01833 | Pado-UA_266 | KP940285 | 2.04 |
| seq25339 | Pado-UA_315 | JN609647.1* | 2.04 |
| seq25159 | Pado-UA_296 | KP940316 | 1.84 |
| seq25703 | Pado-UA_247 | KJ825938* | 1.84 |
| seq25751 | Pado-UA_341 | KP940333 | 1.84 |
| seq23146 | Pado-UA_252 | KJ825943* | 1.64 |
| seq23304 | Pado-UA_290 | KP940310 | 1.64 |
| seq11402 | Pado-UA_277 | KP940297 | 1.64 |
| seq26143 | Pado-UA_351 | KP940343 | 1.64 |
| seq13073 | Pado-UA_280 | KP940300 | 1.43 |
| seq11982 | Pado-UA_278 | KP940298 | 1.43 |
| seq25525 | Pado-UA_333 | KP940325 | 1.43 |
| seq25610 | Pado-UA_337 | KP940329 | 1.23 |
| seq18496 | Pado-UA_285 | KP940305 | 1.02 |
| seq06555 | Pado-UA_273 | KP940292 | 1.02 |
| seq01345 | Pado-UA_264 | KP940283 | 1.02 |
| seq06072 | Pado-UA_271 | KP940290 | 1.02 |
| seq01064 | Pado-UA_263 | KP940282 | 1.02 |
| seq25843 | Pado-UA_344 | KP940336 | 1.02 |
| seq16648 | Pado-UA_255 | KJ825945* | 0.82 |
| seq18621 | Pado-UA_286 | KP940287 | 0.82 |
| seq09776 | Pado-UA_276 | KP940286 | 0.82 |
| seq13489 | Pado-UA_336 | KP940328 | 0.82 |
| seq04799 | Pado-UA_270 | KP940289 | 0.82 |
| seq01198 | Pado-UA_317 | JN609640.1* | 0.82 |
| seq25359 | Pado-UA_335 | KP940327 | 0.82 |
| seq23854 | Pado-UA_293 | KP940313 | 0.61 |
| seq08911 | Pado-UA_275 | KP940295 | 0.61 |
| seq17491 | Pado-UA_282 | KP940302 | 0.61 |
| seq23378 | Pado-UA_291 | KP940311 | 0.61 |
| seq07043 | Pado-UA_274 | KP940293 | 0.41 |
| seq01384 | Pado-UA_265 | KP940284 | 0.41 |
| seq25870 | Pado-UA_345 | KP940337 | 0.41 |
| seq28555 | Pado-UA_348 | KP940340 | 0.41 |
| seq21475 | Pado-UA_288 | KP940308 | 0.20 |

*already published genbank sequences

Supplementary Table 3: MHC alleles (nucleotides) translated in amino acid MHC alleles (AA) and functional MHC alleles (FA)

| **MHC alleles (85)** | **AA translated MHC alleles (78)** | **Functional MHC alleles (59)** | **Frequency of funct. MHC alleles (%)** |
| --- | --- | --- | --- |
| Pado-UA_238, Pado-UA_266, Pado-UA_268, Pado-UA_271, Pado-UA_272, Pado-UA_274, Pado-UA_280, Pado-UA_298, Pado-UA_246, Pado-UA_254, Pado-UA_328, Pado-UA_270, Pado-UA_273, Pado-UA_253, gb\|KC585634.1\|, Pado-UA_299 | AA00217, AA01833, AA04517, AA06072, AA06551, AA07043, AA13073, AA25331, AA25361, AA25416, AA25422 | **FA00217*** | **80.04** |
| Pado-UA_239, Pado-UA_288, Pado-UA_346 | AA21013, AA21475, AA26010 | **FA21013*** | **40.53** |
| Pado-UA_276, Pado-UA_251 | AA09776, AA25566 | **FA09776*** | **30.25** |
| Pado-UA_245 | AA25663 | **FA25663*** | **21.19** |
| Pado-UA_261, Pado-UA_342 | AA00275 | **FA00275*** | **19.96** |
| Pado-UA_286, Pado-UA_248, Pado-UA_349 | AA18621, AA26078, AA26092 | **FA18621*** | **16.26** |
| Pado-UA_290, Pado-UA_244 | AA23304, AA25986 | **FA23304*** | **15.84** |
| Pado-UA_269, Pado-UA_330, Pado-UA_339 | AA25259, AA25459, AA25677 | **FA25259*** | **16.05** |
| Pado-UA_338 | AA25614 | **FA25614*** | **15.64** |
| Pado-UA_343 | AA25835 | **FA25835*** | **12.55** |
| Pado-UA_322 | AA25163 | FA25163 | 9.47 |
| Pado-UA_257 | AA26163 | FA26163 | 8.85 |
| Pado-UA_297 | AA25202 | FA25202 | 8.23 |
| Pado-UA_267 | AA26190 | FA26190 | 8.23 |
| Pado-UA_329 | AA25435 | FA25435 | 8.02 |
| Pado-UA_256 | AA07442 | FA07442 | 7.61 |
| Pado-UA_258 | AA25907 | FA25907 | 6.58 |
| Pado-UA_331 | AA25483 | FA25483 | 6.38 |
| Pado-UA_294 | AA26174 | FA26174 | 5.97 |
| Pado-UA_332 | AA25499 | FA25499 | 5.56 |
| Pado-UA_262 | AA00886 | FA00886 | 5.35 |
| Pado-UA_347 | AA26020 | FA26020 | 4.94 |
| Pado-UA_283, Pado-UA_352 | AA18342, AA26154 | FA18342 | 4.94 |
| Pado-UA_252, Pado-UA_350 | AA23146 | FA23146 | 4.73 |
| Pado-UA_295 | AA25144 | FA25144 | 4.73 |
| Pado-UA_319 | AA25872 | FA25872 | 4.53 |
| Pado-UA_284 | AA18381 | FA18381 | 4.12 |
| Pado-UA_340 | AA25689 | FA25689 | 3.50 |
| Pado-UA_281 | AA13724 | FA13724 | 3.29 |
| Pado-UA_289 | AA26120 | FA26120 | 2.88 |
| Pado-UA_334 | AA25531 | FA25531 | 2.67 |
| Pado-UA_250 | AA25581 | FA25581 | 2.67 |
| Pado-UA_292 | AA23531 | FA23531 | 2.26 |
| Pado-UA_279 | AA12426 | FA12426 | 2.06 |
| Pado-UA_315 | AA25339 | FA25339 | 2.06 |
| Pado-UA_287 | AA25979 | FA25979 | 2.06 |
| Pado-UA_296 | AA25159 | FA25159 | 1.85 |
| Pado-UA_247 | AA25703 | FA25703 | 1.85 |
| Pado-UA_341 | AA25751 | FA25751 | 1.85 |
| Pado-UA_277 | AA11402 | FA11402 | 1.65 |
| Pado-UA_351 | AA26143 | FA26143 | 1.65 |
| Pado-UA_278 | AA11982 | FA11982 | 1.44 |
| Pado-UA_333 | AA25525 | FA25525 | 1.44 |
| Pado-UA_337 | AA25610 | FA25610 | 1.23 |
| Pado-UA_263 | AA01064 | FA01064 | 1.03 |
| Pado-UA_264 | AA01345 | FA01345 | 1.03 |
| Pado-UA_285 | AA18496 | FA18496 | 1.03 |
| Pado-UA_344 | AA25843 | FA25843 | 1.03 |
| Pado-UA_317 | AA01198 | FA01198 | 0.82 |
| Pado-UA_336 | AA13489 | FA13489 | 0.82 |
| Pado-UA_255 | AA16648 | FA16648 | 0.82 |
| Pado-UA_335 | AA25359 | FA25359 | 0.82 |
| Pado-UA_275 | AA08911 | FA08911 | 0.62 |
| Pado-UA_282 | AA17491 | FA17491 | 0.62 |
| Pado-UA_291 | AA23378 | FA23378 | 0.62 |
| Pado-UA_293 | AA23854 | FA23854 | 0.62 |
| Pado-UA_265 | AA01384 | FA01384 | 0.41 |
| Pado-UA_345 | AA25870 | FA25870 | 0.41 |
| Pado-UA_348 | AA28555 | FA28555 | 0.41 |
| *only the presence/absence (1/0) data of the 10 most common functional MHC alleles were included in the analysis | | | |

Supplementary Table 4: ESR (linear mixed model)

| Model-averaged coefficients | Estimate | Std. Error | p value | RVI |
| --- | --- | --- | --- | --- |
| (Intercept) | 0.70556 | 0.31662 | 0.0266 |  |
| **FA09776** | -0.10733 | 0.04213 | 0.0116 | **0.87** |
| FA21013 | -0.09546 | 0.06013 | 0.1148 | 0.58 |
| Number of funct. MHC alleles | 0.03308 | 0.02152 | 0.1267 | 0.56 |
| Condition | 0.02273 | 0.01406 | 0.1092 | 0.55 |
| Microsatellite heterozygosity | -0.44880 | 0.32767 | 0.1750 | 0.45 |
| Sex | -0.05175 | 0.03880 | 0.1866 | 0.44 |
| Clutch number | -0.02540 | 0.02249 | 0.2635 | 0.38 |

Supplementary Table 5: HCT (linear mixed model)

| Model-averaged coefficients | Estimate | Std. Error | p value | RVI |
| --- | --- | --- | --- | --- |
| (Intercept) | 0.44255 | 0.06298 | <2e-16 |  |
| **FA09776** | 0.02124 | 0.00952 | 0.0271 | **0.79** |
| Condition | -0.00578 | 0.00305 | 0.0602 | 0.66 |
| Clutch number | 0.00810 | 0.00494 | 0.1045 | 0.55 |
| FA23304 | -0.01753 | 0.01128 | 0.1237 | 0.50 |
| Microsatellite heterozygosity | 0.09085 | 0.07386 | 0.2233 | 0.41 |
| Number of funct. MHC alleles | -0.00268 | 0.00319 | 0.4046 | 0.32 |
| Sex | -0.00442 | 0.00828 | 0.5970 | 0.27 |

Supplementary Table 6: BKA (linear mixed model)

| Model-averaged coefficients | Estimate | Std. Error | p value | RVI |
| --- | --- | --- | --- | --- |
| (Intercept) | 0.15108 | 0.22903 | 0.5095 |  |
| **FA09776** | -0.12772 | 0.05028 | 0.0111 | **0.88** |
| **FA25663** | 0.14289 | 0.05600 | 0.0107 | **0.87** |
| Sex | 0.05638 | 0.04391 | 0.1992 | 0.42 |
| Number of funct. MHC alleles | 0.02214 | 0.01944 | 0.2546 | 0.40 |
| Clutch number | -0.01599 | 0.02588 | 0.5368 | 0.28 |
| Microsatellite heterozygosity | -0.17739 | 0.40643 | 0.6625 | 0.26 |
| Condition | -0.00422 | 0.01605 | 0.7924 | 0.25 |

Supplementary Table 7: H/L ratio (linear mixed model)

| Model-averaged coefficients | Estimate | Std. Error | p value | RVI |
| --- | --- | --- | --- | --- |
| (Intercept) | -1.19277 | 0.37681 | 0.0016 |  |
| **FA00217** | 0.16264 | 0.06991 | 0.0200 | **0.82** |
| FA00275 | 0.13187 | 0.06812 | 0.0529 | 0.68 |
| Number of funct. MHC alleles | 0.03217 | 0.01992 | 0.1064 | 0.51 |
| Sex | 0.08183 | 0.05379 | 0.1282 | 0.51 |
| Microsatellite heterozygosity | 0.47634 | 0.48832 | 0.3293 | 0.35 |
| Clutch number | -0.00428 | 0.03359 | 0.8986 | 0.25 |
| Condition | 0.00325 | 0.01976 | 0.8695 | 0.25 |

Supplementary Table 8: PHA (11 day old nestlings) (linear mixed model)

| Model-averaged coefficients | Estimate | Std. Error | p value | RVI |
| --- | --- | --- | --- | --- |
| (Intercept) | 0.41419 | 0.18145 | 0.0225 |  |
| **FA09776** | -0.09554 | 0.04141 | 0.0211 | **0.82** |
| FA25614 | -0.08507 | 0.04818 | 0.0774 | 0.61 |
| Clutch number | -0.02579 | 0.02060 | 0.2105 | 0.42 |
| Condition (11 days) | -0.00587 | 0.00777 | 0.4500 | 0.30 |
| Microsatellite heterozygosity | 0.17736 | 0.31597 | 0.5746 | 0.28 |
| Number of funct. MHC alleles | 0.00433 | 0.01421 | 0.7608 | 0.27 |
| Sex | 0.00591 | 0.03321 | 0.8588 | 0.25 |

Supplementary Table 9: PHA (1st adult response) (linear mixed model)

| Model-averaged coefficients | Estimate | Std. Error | p value | RVI |
| --- | --- | --- | --- | --- |
| (Intercept) | 0.87736 | 0.47757 | 0.0691 |  |
| Condition | -0.02991 | 0.02051 | 0.1532 | 0.45 |
| Microsatellite heterozygosity | -0.71822 | 0.49647 | 0.1566 | 0.44 |
| Number of funct. MHC alleles | 0.01164 | 0.02215 | 0.6067 | 0.26 |
| Clutch number | 0.00737 | 0.03825 | 0.8503 | 0.24 |
| Sex | 0.01225 | 0.05895 | 0.8387 | 0.24 |

Supplementary Table 10: PHA2 (2nd adult response) (linear mixed model)

| Model-averaged coefficients | Estimate | Std. Error | p value | RVI |
| --- | --- | --- | --- | --- |
| (Intercept) | 1.68970 | 0.63897 | 0.0088 |  |
| **Number of funct. MHC alleles** | -0.08930 | 0.02351 | 0.0002 | **0.99** |
| **FA23304** | -0.26406 | 0.10568 | 0.0144 | **0.85** |
| Clutch number | -0.07380 | 0.04052 | 0.0747 | 0.59 |
| Microsatellite heterozygosity | -1.00080 | 0.55154 | 0.0757 | 0.59 |
| Sex | 0.06259 | 0.06674 | 0.3588 | 0.30 |
| Condition | -0.01338 | 0.02388 | 0.5830 | 0.25 |

Supplementary Table 11: SRBC (linear mixed model)

| Model-averaged coefficients | Estimate | Std. Error | p value | RVI |
| --- | --- | --- | --- | --- |
| (Intercept) | 7.22436 | 3.70190 | 0.0545 |  |
| **FA18621** | 2.80550 | 1.00813 | 0.0064 | **0.93** |
| Number of funct. MHC alleles | -0.43098 | 0.25638 | 0.0997 | 0.55 |
| Clutch number | -0.43714 | 0.37396 | 0.2528 | 0.36 |
| Microsatellite heterozygosity | 4.21318 | 5.85667 | 0.4814 | 0.27 |
| Condition | -0.04298 | 0.25577 | 0.8694 | 0.23 |
| Sex | 0.10342 | 0.74187 | 0.8914 | 0.23 |

**Supplementary literature**

Bonneaud, C., Richard, M., Faivre, B., Westerdahl, H. & Sorci, G. (2005) An *Mhc* class I allele associated to the expression of T-dependent immune response in the house sparrow. *Immunogenetics,* **57,** 782-789.

Campbell, T.W. (1995) *Avian Hematology and Cytology,* 2nd Edition edn. Iowa State Univ. Press, Ames, Iowa.

Dawson, D.A., Horsburgh, G.J., Krupa, A.P., Stewart, I.R.K., Skjelseth, S., Jensen, H., Ball, A.D., Spurgin, L.G., Mannarelli, M.E., Nakagawa, S., Schroeder, J., Vangestel, C., Hinten, G.N. & Burke, T. (2012) Microsatellite resources for Passeridae species: a predicted microsatellite map of the house sparrow *Passer domesticus*. *Molecular Ecology Resources,* **12,** 501-523.

Galan, M., Guivier, E., Caraux, G., Charbonnel, N. & Cosson, J.F. (2010) A 454 multiplex sequencing method for rapid and reliable genotyping of highly polymorphic genes in large-scale studies. *BMC Genomics,* **11,** 296.

Griffith, S.C., Dawson, D.A., Jensen, H., Ockendon, N., Greig, C., Neuman, K. & Burke, T. (2007) Fourteen polymorphic microsatellite loci characterized in the house sparrow *Passer domesticus* (Passeridae, Aves). *Molecular Ecology Notes,* **7,** 333-336.

Griffith, S.C., Stewart, I.R.K., Dawson, D.A., Owens, I.P.F. & Burke, T. (1999) Contrasting levels of extra-pair paternity in mainland and island populations of the house sparrow (*Passer domesticus*): is there an ‘island effect’? *Biological Journal of the Linnean Society,* **68,** 303-316.

Liebl, A.L. & Martin II, L.B. (2009) Simple quantification of blood and plasma antimicrobial capacity using spectrophotometry. *Functional Ecology,* **23,** 1091-1096.

Neuman, K. & Wetton, J.H. (1996) Highly polymorphic microsatellites in the house sparrow *Passer domesticus*. *Molecular Ecology,* **5,** 307-309.

Pap, P.L., Vágási, C.I., Czirják, G.Á. & Barta, Z. (2008) Diet quality affects postnuptial molting and feather quality of the house sparrow (*Passer domesticus*): Interaction with humoral immune function? *Canadian Journal of Zoology,* **86,** 834-842.

Richardson, D.S., Jury, F.L., Dawson, D.A., Salgueiro, P., Komdeur, J. & Burke, T. (2000) Fifty Seychelles warbler (*Acrocephalus sechellensis*) microsatellite loci polymorphic in Sylviidae species and their cross-species amplification in other passerine birds. *Molecular Ecology,* **9,** 2226-2231.

Schalm, O.W., Jain, N.C. & Carroll, E.A. (1975) *Veterinary Hematology*. Lea & Febiger, Saint Louis.

Sepil, I., Lachish, S. & Sheldon, B.C. (2013) *Mhc*-linked survival and lifetime reproductive success in a wild population of great tits. *Molecular Ecology,* **22,** 384-396.

Tella, J.L., Lemus, J.A., Carrete, M. & Blanco, G. (2008) The PHA test reflects acquired T-cell mediated immunocompetence in birds. *PLoS ONE,* **3,** e3295.

Vinkler, M., Bainova, H. & Albrecht, T. (2010) Functional analysis of the skin-swelling response to phytohaemagglutinin. *Functional Ecology,* **24,** 1081-1086.
